# Flood risk assessment of the Garita River in the urban zone of San Luis Potosí City, by hydrodynamic modeling

**DOI:** 10.1038/s41598-024-66743-1

**Published:** 2024-07-10

**Authors:** Katya Onchi-Ramos, Clemente Rodríguez-Cuevas, Carlos Couder-Castañeda, Diego-Alfredo Padilla-Pérez

**Affiliations:** 1 Universidad Autónoma de San Luis Potosí, Facultad de Ingeniería, 79290 San Luis Potosí, SLP Mexico; 2 Instituto Politénico Nacional, Centro de Desarrollo Aeroespacial, Mexico City, 06610 Mexico

**Keywords:** Hydrodynamics, River, Droughts, Numerical modeling, Model, Environmental impact, Hydrology

## Abstract

Rapid and uncontrolled urban growth and land use changes in watersheds worldwide have led to increased surface runoff within metropolitan areas, coupled with climate change, creating a risk for residents during the rainy season. The city of San Luis Potosí is no exception to this phenomenon. One affected watercourse is the Garita Stream, which flows inside the city near urbanization. It is essential to analyze the effects of urban sprawl on this stream based on historical precipitation data for the town. Hydrological and topographical information were required to conduct this research. The hydrological study of the basin involved analyzing the region’s geomorphology and historical climatological data. For the stream’s topography, aerial photogrammetry using an unmanned aerial Vehicle (UAV) and Global Navigation Satellite System (GNSS) equipment was employed to conduct topographic surveys in the area. To find out when the Garita stream would overflow and which areas are most likely to flood, numerical modeling was done using 1D, 2D, and 3D programs like SWMM5 (Storm Water Management Model), HEC-RAS (Hydrologic Engineering Center’s River Analysis System), and EDFC Explorer (Environmental Fluid Dynamics Code). These models simulated different return periods and their correlation with current flooding events recorded in the area, thereby further proposing solutions to mitigate overflow issues. By conducting these simulations and analyzing the results, solutions can be suggested to address the overflow problems in the area based on historical flood events at various return periods caused by the Garita Stream.

## Introduction

One of the problems faced by populations in different parts of the world is flooding due to a variety of natural factors, such as the relationship between rainfall and runoff, which is becoming more intense due to climate change, and the change in land use due to population growth, which generates more runoff on impervious surfaces^1^. Proof of this can be found in the city of San Luis Potosi, where in August 2016, atypical rainfall occurred in the southeastern area of the town, where the Garita stream is located, causing it to overflow and, consequently, flooding the main avenues of the capital of San Luis Potosi, endangering the lives of pedestrians walking in the area^2^.

The behavior of the water cycle in an urbanized watershed is altered by the waterproof cover created in the area. For this reason, government agencies have carried out studies to facilitate the location of critical points where flooding can occur, with the study area being a vital flood zone and an essential area of the city due to its malls and densely populated areas^3^.

The city’s flood atlas shows the different areas prone to flooding, including the Garita Stream area. The most relevant problem of flooding in the southwestern area of the city is the poor planning of urban growth since the invasion of the mainstream of the basin, the Garita stream, has not been fully considered, directly affecting the residential settlements along the entire stream^4^.

The infrastructure developed in urban areas impermeabilizes areas previously covered by vegetation and natural soil, concentrates the population in small areas, and uncontrollably expands the periphery of cities, resulting in a modification of the hydrological water cycle that directly affects the response of runoff, infiltration, and climate in the area where urbanization is established. Nevertheless, urban growth seems permanent, including strip malls and corporate campuses, covering and encroaching on natural areas, waterways, and floodplains, intensifying and accelerating rainfall response to runoff.

The impervious surface of the cities is a factor that increases the runoff coefficient, which increases the flow velocity and cost, forcing the population to work to protect the inhabitants and their property from flood damage^5^. The United Nations Office for Disaster Risk Reduction mentions that in the last 20 years, there have been twice as many significant floods, from 1384 to 3254, while storms increased from 1457 to 2034. These are the most produced compared to other natural disasters^6^. According to the International Disaster Database, several floods were recorded in Mexico from 1990 to 2020, affecting 4,532,336 inhabitants in different states of México. The study of flooding in an urbanized area requires different analyses of existing data and the generation of missing data within the watershed under investigation due to the population growth that has occurred in recent years. The change in land use has made the watershed almost impervious, causing an imbalance in the hydrological cycle.

To understand why flooding occurs in different areas near the creek, different software models were applied to simulate the effects of flooding in the urbanization zones using existing and estimated data^7,8^. The behavior of the hydrological cycle in an urbanized watershed is altered by the impermeable cover generated in the area. The increase in San Luis Potosí has caused significant impacts due to land use changes, resulting in increased flooding in different city areas with every rainfall. As a result, government agencies have conducted studies to locate critical points where floods can occur more easily. The study area is a vital flood zone and an important place in the city due to its malls and densely populated areas. The most relevant problem regarding the floods generated in the southwestern area of the city is poor urban growth planning, as the complete invasion of the mainstream of the watershed has not been adequately considered. This directly affects residential settlements along the entire course of the Garita stream. In the development process, it is necessary to consider the stream’s maintenance and implementing systems that allow diversion or capture of the runoff to minimize downstream issues. This also goes hand in hand with raising awareness among the population about the proper use and management of the stream and nearby drainage systems. According to the latest update in the 2012 flood atlas for the area, considering the population growth upstream in the watershed, it is a flood-prone area that requires an update due to ongoing urban expansion and significant flooding events^9^. In 2016, the Garita stream exceeded its hydraulic capacity, resulting in overflow and water flowing towards Chapultepec and Salvador Nava Avenues. According to reports, the water was powerful enough to sweep away a pedestrian attempting to cross the avenue under a public bus, highlighting the importance of studying the stream’s behavior in the area. Although San Luis Potosí is not one of the states of Mexico most affected by floods, the city’s urban growth has resulted in risk areas, as occurred in 2016, requiring a diagnosis to determine the current surface conditions and possible overflows in the same zone. San Luis Potosí is located in the center of the Mexican Republic and is composed of the San Luis Potosí and Soledad de Graciano Sánchez municipalities. It has an approximate area of 200 $\hbox {km}^2$ and a population of nearly 1,000,000 inhabitants.

The city generally has a dry climate in the south and a dry-semi-hot environment in the north. The average annual temperature is $16.8\,\,^{\circ }$C, with a maximum of $35\,\,^{\circ }$C and a minimum of $7\,\,^{\circ }$C. Warm temperatures are experienced from March to October, while colder temperatures occur from November to February. The average annual precipitation is 372.9 mm.

The research subject, the Garita Stream, is located southwest of San Luis Potosí. It has a length of approximately 7 km from its basin to its outlet in the Tenería dam, covering an area of about 0.13 $\hbox {km}^2$ within Tangamanga I Park. Tangamanga I Park is considered the lungs of the city and one of the country’s largest and most significant parks, with an area of 4.2 $\hbox {km}^2$, more extensive than New York’s Central Park (3.4 $\hbox {km}^2$). The park is also self-sustainable, as it has two treatment plants for irrigation and compost generation for its vegetation.

The Garita Stream watershed is an urban watershed, as the population has encroached on a significant portion of its area. The watershed has an area of 8.4 $\hbox {km}^2$, according to the risk atlas by CENAPRED and the city’s municipal government. The population within the site is approximately 35,000 inhabitants.

The main objective of this research is to assess the Garita Stream within the city of San Luis Potosí and determine the conditions under which the stream overflows. The EFDC (Environmental Fluid Dynamics Code) model is a powerful tool for simulating fluid flow in natural water bodies^10–13^. Still, it can also be adapted for urban flooding simulations^14–18^. For this reason, it was the primary tool for performing the simulations.

To outline an approach using EFDC for simulating urban flooding, it is necessary to set up a model domain to encompass the metropolitan area under study. This involves defining the domain’s boundaries, specifying the area’s bathymetry/topography, and identifying critical features such as roads, buildings, drainage networks, etc. Subsequently, determine the sources of water input into the system. This could include rainfall data, runoff from impervious surfaces such as roads and pavements, contributions from stormwater drainage systems, and any other relevant sources.

Determining the hydraulic properties of different land covers within the urban area is necessary to specify parameters like roughness coefficients for various types of surfaces (pavement, grass, buildings, etc.), infiltration rates, and hydraulic conductivity for porous surfaces.

Definitions of boundary conditions in the model domain must include water levels at the boundaries, inflow and outflow rates at inlets and outfalls, and any other relevant boundary constraints. The model has to be calibrated using observed data from past flooding events or other appropriate sources. This might involve adjusting the model parameters to match observed water levels or flow velocities. Validation involves comparing model predictions to independent datasets to assess their accuracy and reliability. It is necessary to run the EFDC model several times with specified inputs, boundary conditions, and calibrated parameters. The model will simulate water movement through the urban area over time, allowing you to observe how flooding develops and propagates under different scenarios. Finally, to analyze the simulations and understand the dynamics of urban flooding in your study area, this could include generating flood maps to identify vulnerable areas, assessing the impact of different interventions on flood risk, and exploring potential mitigation strategies.

## Material and methods

### Description of the study area

**Figure 1:**
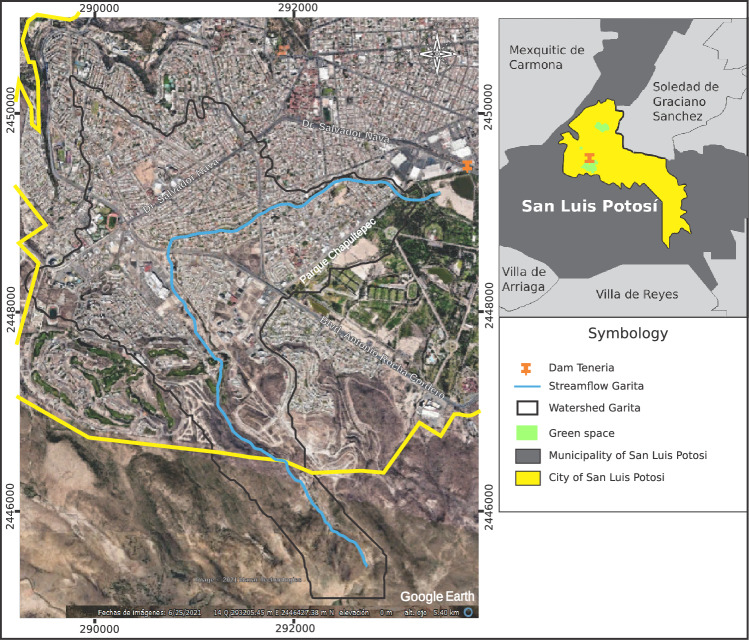
Location of the Garita Stream. Image modified with free QGIS software version 3.34 LTR Win (https://www.qgis.org/en/site/).

The municipality of San Luis Potosí is located in the state of San Luis Potosí in the central highlands of the Mexican Republic, representing 3.12% of the national territory; has an approximate area of 200 $\hbox {km}^2$, with a population of almost 1,000,000 inhabitants^19^. The Garita stream, which is the subject of this study, is located in the southwestern area of the capital of the state of Potosí, approximately 7 km long from the beginning of its watershed to its mouth at the La Tería dam, with about 0.13 $\hbox {km}^2$ within the Tangamanga I Park. Figure 1 shows the location of the Garita stream within the city.

### Geological composition of the area

The city of San Luis Potosí is located on a soil known as the Volcanic Field, with the majority of this soil being Aluvial and Rhyolite-Tuff, which are extrusive igneous rocks. Generally, the soil has a medium to low permeability, with higher permeability in the Alluvial zone in non-urbanized areas^20^.

**Figure 2:**
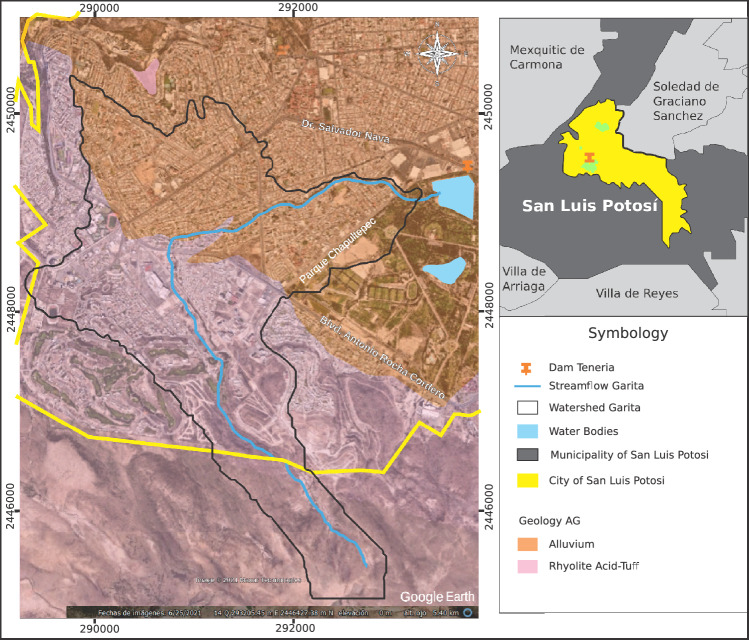
Characterized geology of the Garita basin. Image modified with free QGIS software version 3.34 LTR Win (https://www.qgis.org/en/site/).

The Rhyolite soil in the Garita Stream basin covers approximately 57% of its area, as seen in Fig. 2. The basin is mainly filled with Quaternary sediments in the most urbanized zone and Neogene sediments upstream, where a large part of the urban area is also located^21^.

The thickness of the Quaternary deposits in San Luis Potosí varies greatly and is influenced by the shape of the bedrock, ranging from 50 to over 500 m.

### Edaphology of the zone

The primary soils in San Luis Potosí are semi-desert and desert types known as Regosol, Planosol, and Xenosol. The first two are the predominant soils in the basin.

The first two are lithosols, which have a light-colored surface layer due to low organic matter content, while the third soil type is shallow and derived from residual rocks of the rhyolitic type. In the mountainous area, rocky outcrops contribute to the soils’ medium permeability in non-urbanized regions upstream of the basin.

**Figure 3:**
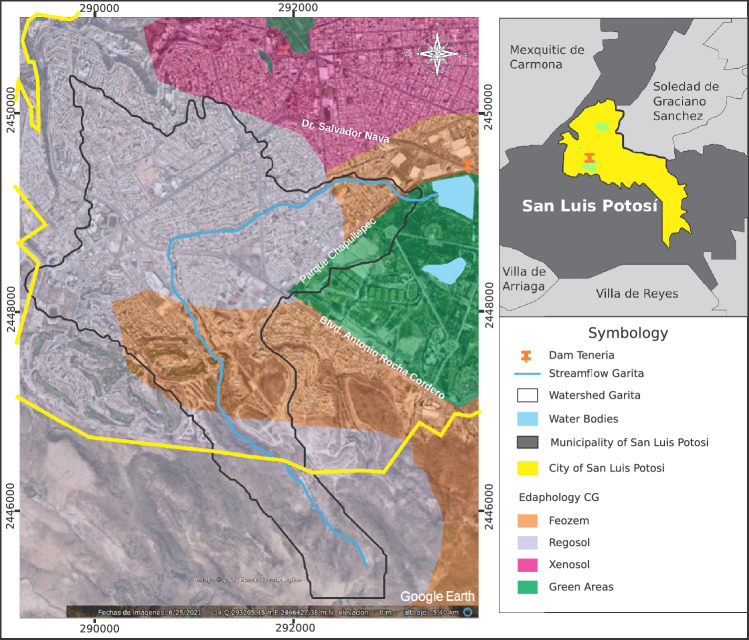
Edaphology of the Garita basin. Image modified with free QGIS software version 3.34 LTR Win (https://www.qgis.org/en/site/).

The basin exhibits typical soil characteristics of an arid climate, classified as regosol, covering approximately 74% of the basin area (see Fig. 3). The soil is considered poor in organic matter and is superficial and residual^22^.

### Orography of the zone

**Figure 4:**
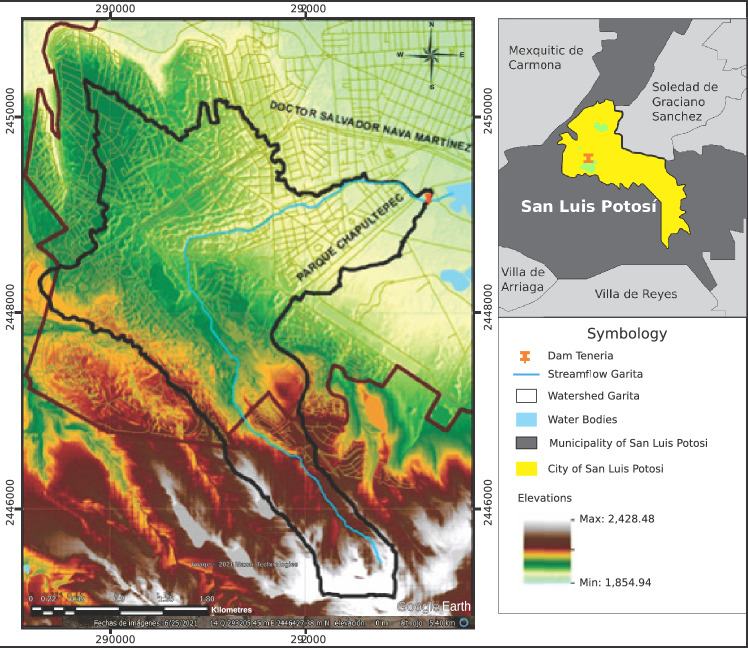
Characterized Orography of the Garita basin. Image modified with free QGIS software version 3.34 LTR Win (https://www.qgis.org/en/site/).

The shape of the terrain results from weathering, climate, runoff, and geological processes that shape the soil’s surface and promote the diversity of landforms. In a developing city like San Luis Potosí, the shape of the terrain has been modified by urbanization. The Garita basin is a mountainous area with xerophytic vegetation, with altitudes ranging from 1888 to 2234 m above sea level (see Fig. 4).

### Hydrography

**Figure 5:**
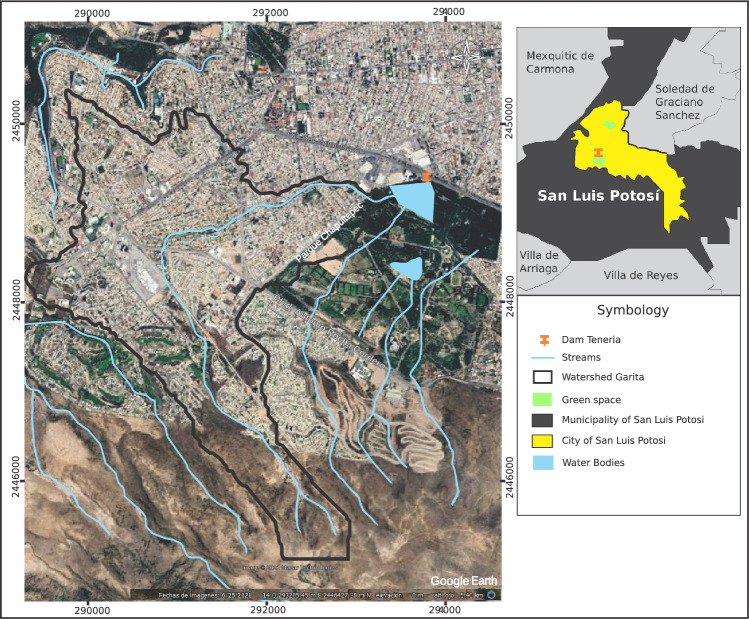
Hydrography of the Garita basin. Image modified with free QGIS software version 3.34 LTR Win (https://www.qgis.org/en/site/).

The municipality of San Luis and its conurbation, with the code RH37, is located in the southern portion of the El Salado hydrological region. Specifically, the studied stream basin is within the Presa San José-Los Pilares and others. Within the Presa San José-Los Pilares and other basins (RH37G), there is the micro-basin of Garita, which is of the exoreic type with disordered drainage due to the urbanization of the sub-basin. The main watercourse in the area of interest is the Garita Stream, which has an approximate length of 7 km (see Fig. 5)^23–25^.

### Wheater of the zone

**Figure 6:**
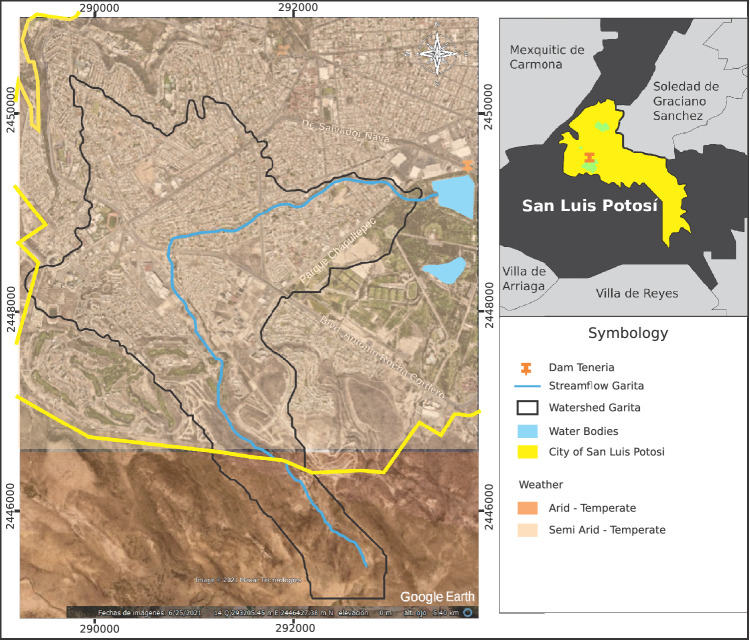
The climate in the Garita Basin. Image modified with free QGIS software version 3.34 LTR Win (https://www.qgis.org/en/site/).

The city’s climate is generally arid-temperate, belonging to group B according to Köppen, with an average annual temperature between 12 and 18$\,\,^{\circ }$C, and in the coldest month, between − 3 and $18\,\,^{\circ }$C^26^. In the municipality, the annual average is $16.8\,\,^{\circ }$C, and the average for the month of maximum temperature is $21.5\,\,^{\circ }$C, with a maximum of $35\,\,^{\circ }$C and an average for the month of minimum temperature of $12.9\,\,^{\circ }$C and absolute minimum of $7\,\,^{\circ }$C. The warm temperature is from March to October, and cold from November to February. Within the basin, the dominant climate is the same as within the city, covering an area of approximately 87%; in a smaller percentage, the climate is semi-arid temperate (see Fig. 6), with average annual temperature and the coldest month equal to the arid environment. Inside the city. There are heavy rains in summer and some in winter, with an average annual precipitation (APM) of 386 mm, according to data from 1949 to 2018^27^.

## Model setup

According to their origin, floods can be classified as:

- Pluvial floods. They are a consequence of precipitation and occur when the ground has saturated; the surplus accumulates. Its characteristic is that the collected water is precipitated in the area and not from another part of the basin.
- River floods. They are generated when the water from the rivers overflows and remains on the surface of the land near them. In this type of flood, the water overflows on the adjacent land corresponds to rainfall from any part of the basin, not necessarily from the affected area.
- Coastal flooding. These are times when the average sea level decreases due to the tide, allowing it to penetrate inland in coastal areas, covering large land areas. The wave can be generated by cyclone winds over the sea surface and the decrease in minimum pressure.

Another categorization of floods is based on the basin’s response time, which is influenced by its physiographic attributes. This classification consists of two distinct groups: gradual floods, characterized by their extended formation period and resulting primarily in material damages, and rapid floods, which develop swiftly and predominantly result in human casualties within densely inhabited regions.

### Measurement campaigns

The measurement campaigns for the generation of the topography of the Garita stream and surrounding areas were carried out using aerial photogrammetry and GNSS receivers, which obtained, among other things, a precision of centimeters, cartographic details of the area, georeferencing and a shorter execution time.

Before the topographical survey of the stream, a study of the stream was first carried out to observe its conditions in terms of the urban infrastructure around it. Fifteen flights were planned at a height of 100 m, covering the entire urbanized stream and its mouth at the *La Teneria* dam, verifying that the previous and following ones overlap from start to finish.

The static survey of control points was carried out on different dates according to the availability to use them, so the GPS was left on for around 45 min at each end, measuring all the points in 4 days, while the GNSS was left on for 15 min at the first point and ten at the following ones, counting all the points in two days. Simultaneously, with the measurements of the Promark3 equipment, the UAV took approximately 15 min to verify that the battery was sufficient to carry out the determined area. The urbanization process has led to infrastructure development along the stream, which is considered significant due to its importance. Additionally, measurement campaigns were conducted to determine certain bridges’ locations and dimensions accurately. The data acquisition utilizing the Promark3 equipment was conducted over distinct days due to the operational constraints of the receiver.

**Figure 7:**
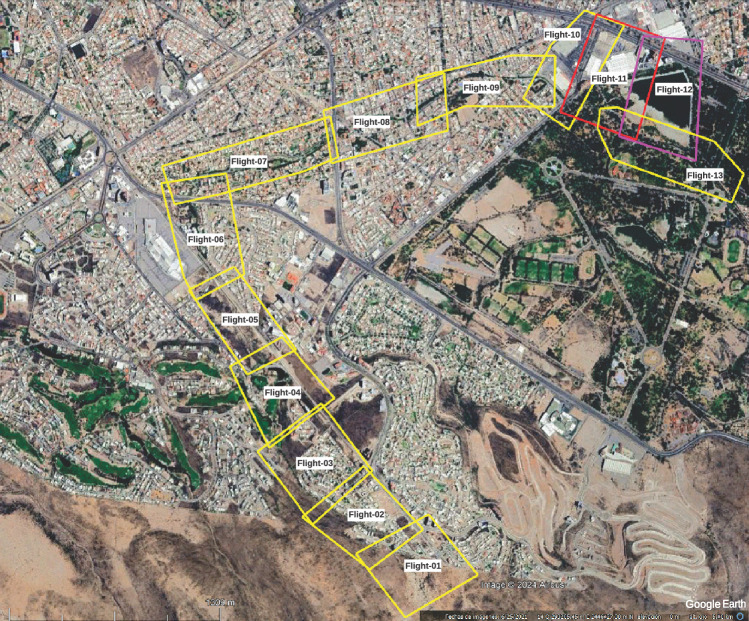
Area covered by each drone flight. The image was made with Google Earth and modified with Inkscape (inkscape.com).

The flights carried out by the UAV were planned using the DroneDeploy application, taking care that there was an overlap between the images captured by the camera of between 60 and 80% longitudinal and lateral. As it was impossible to cover the entire length of Arroyo Garita with a solo flight, it was necessary to make 13 flights. Each flight was limited to 15 min due to the autonomy of its battery and covered an approximate area of 0.25 $\hbox {km}^2$. The flights were carried out on the following dates, shown in Table 1. Figure 7 displays each flight’s area.

Once the UAV camera captures the images, they are corrected to compensate for lens distortions, lighting variations, etc., aligned, and pasted using the Agisot Metashape software. Control points on the ground must be identified and marked, which the UAV must visualize. Drawing a lime cross on the ground designates the points. The control points were measured using a Trimble R8s differential GPS antenna (DGPS). These points help georeference the images. GPS provides the latitude, longitude, and elevation of the checkpoint. First, generating the point cloud to create the digital elevation models is necessary. To do this, measurement algorithms are used to find common points in the overlapping images using the algorithms proposed by the Agisoft software. A 3D point cloud is generated from the identified common points representing the terrain surface. The 3D point cloud creates a model that includes all objects on the ground, such as buildings and vegetation. This model is known as a digital elevation model.

**Table 1:** Flights and dates when they took place.

Flights	Date
1, 2	16/01/2020
3, 4, 5, 6, 7, 8	21/01/2020
9,10,11,12	22/01/2020
13	25/01/2020

**Figure 8:**
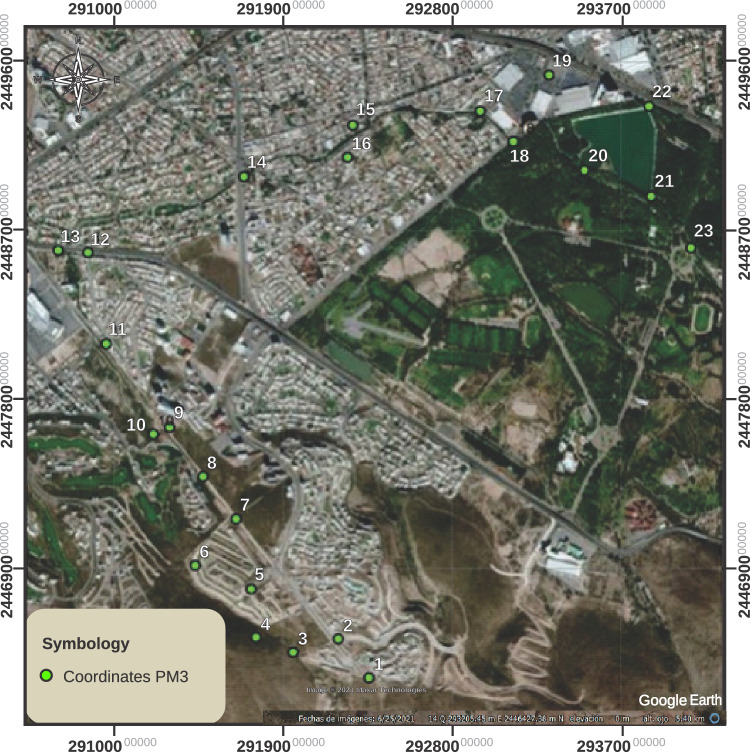
Control points for georeferenced drone flights.

**Figure 9:**
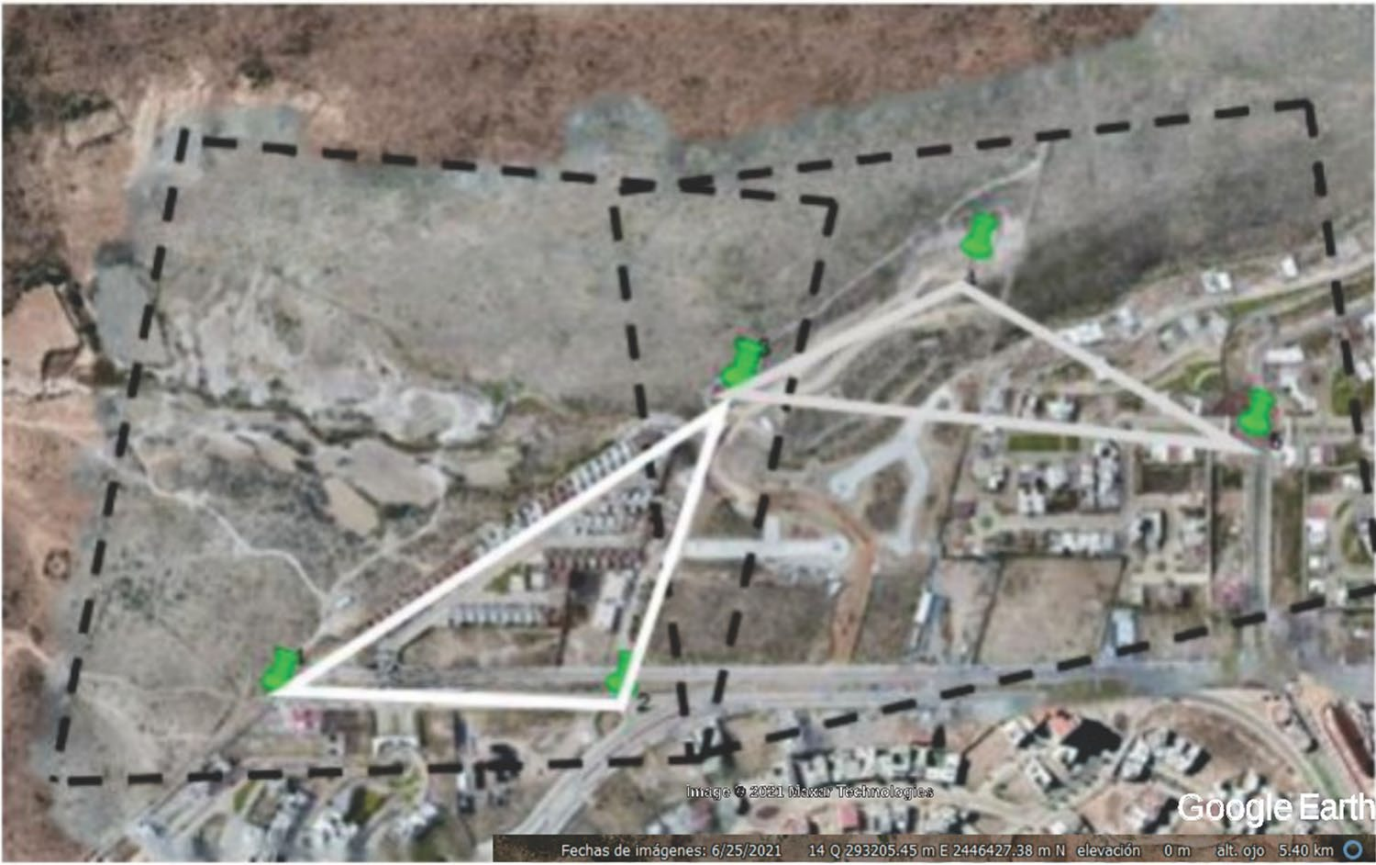
Triangulation in photogrammetric flights.

These activities led to allocating control points, as shown in Fig. 8. Once the flights were planned, the control points were located, forming triangulations within each flight and some overlapping the flights to join the flights, as shown in Fig. 9.

### Devices used and accuracy of data acquisition

A Phantom 4 Pro-UAV captures aerial photos. This UAV is designed for professional use. It is a micro quadcopter type with a weight of 1388 kg. It records 4K videos and captures 20 MP photos. It has obstacle detection in 5 directions, a mechanical shutter that eliminates distortions due to movement in flight, and a sensor that allows you to capture better details of the terrain for the image. Depending on weather conditions, its battery can last up to 30 min in flight.

The horizontal or planimetric error in *x* and *y* of these devices can be from a few centimeters to several decimeters, depending on the quality of the GPS control points used. The altimetric error is usually more significant than the planimetric error. Depending on the GPS’s accuracy and processing quality, it can vary from a few centimeters to a meter. If the processing is high precision and there is good planning (differential GPS, high-resolution camera, advanced processing), the errors can be: Horizontal: 2–5 cm, Vertical: 5–10 cm. If the processing is medium-precision and there is good planning (differential GPS, high-resolution camera, advanced processing), the errors can be: Horizontal: 10–20 cm Vertical: 20–50 cm In this work, medium-precision processing was performed.

**Figure 10:**
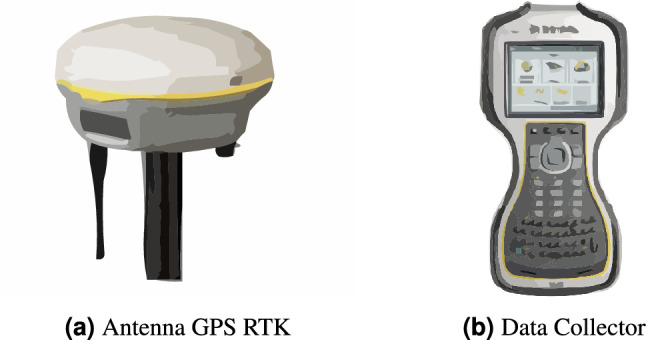
GPS data collector.

A Trimble R8s GPS antenna was used to measure the control points (see Fig. 10). This antenna is an advanced GNSS receiver that provides a point’s position using various positioning technologies and methodologies.

The Trimble R8s is a GPS antenna that can receive signals from multiple GNSS (Global Navigation Satellite Systems) satellite constellations, such as GPS (USA), GLONASS (Russia), Galileo (Europe), and BeiDou (China). This can improve signal accuracy and availability. The receiver triangulates signals from at least four satellites to determine its position. The differences in signal arrival times are used to calculate the distances to each satellite. As the satellites are in motion, the receiver applies error corrections, such as differential correction (DGPS), to improve accuracy. This requires data on the satellite’s location when the antenna collected the geopositioning information. The National Institute of Statistics and Geography (INEGI) has a set of permanently operating fixed stations or antennas called the Active National Geodetic Network, which records data from the Global Navigation Satellite System (GNSS). The antennas are strategically distributed throughout the national territory. They allow the National Geodetic System to be materialized in its horizontal aspect and provide geodetic positioning services to users through online data coordinates of the highest positional accuracy in the country. The Trimble antenna remains at the point for approximately 10 min, allowing it to collect GNSS data and calculate its position through post-processing. Once the information from the control points is obtained, the Trimble Business Center software processes and interprets the GNSS data signals received by the Trimble antenna and the INEGI Active National Geodetic Network antennas.

## Hydrodynamic modeling of the Garita stream

The advancement of numerical and computational models has facilitated numerical modeling to offer comprehensive insights into flood risk and urban planning. By employing this method, assessing the urban flooding reaction triggered by storms becomes feasible to minimize harm and decrease potential hazards. Investigating many physical phenomena necessitates numerical simulations, which rely on observations, discussions, and high-performance computing resources. These simulations enable the analysis of impractical processes to study through experimental means^28^.

In certain instances, it may be necessary to modify the mathematical model by adjusting the parameters or altering the equations to accurately depict the fluid’s behavior near the surface.

**Figure 11:**
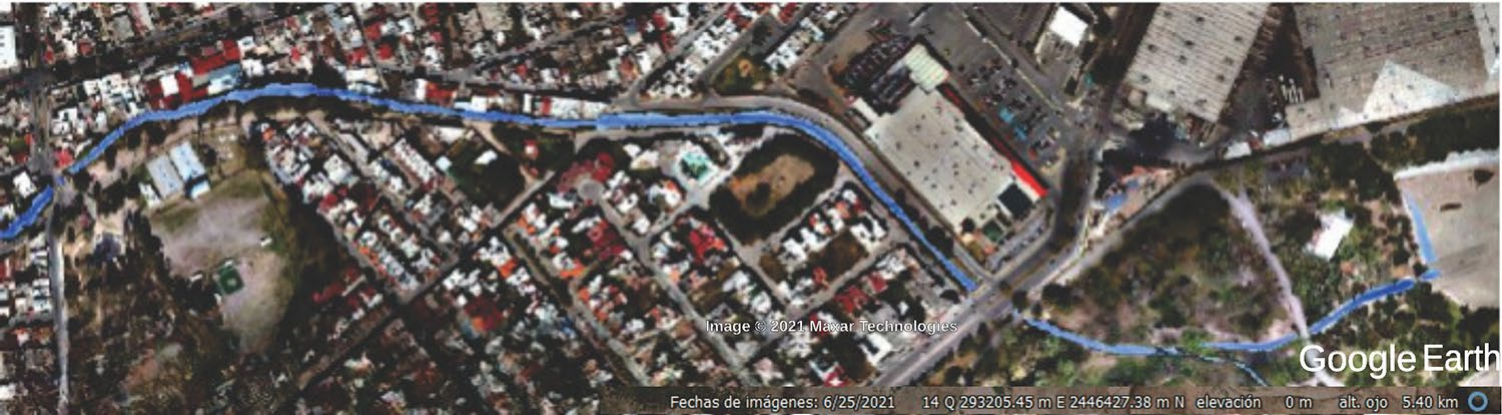
Flood area projection using the HEC-RAS 6.5 Win (https://www.hec.usace.army.mil/software/hec-ras/). Image generated with the HEC-RAS Visualizer.

The HEC-RAS model was used to perform the previous 2D simulations. The sections were built manually for the stream, so they did not intersect and were exported correctly. The sections were made approximately every 50 m on each bridge or important structure, before and after them, for their subsequent implementation within the program. Figure 11 shows the flood area projection generated with HEC-RAS.

Although the results projected in ArcMap are consistent, they show some water stains in the urban area when the water inside the stream has not yet overflowed. This can be solved through more sections or a finer mesh. Due to the limitations of the HEC-RAS model for better resolution, the EFCD Model was used.

EFDC Explorer is a pre-and post-processor for three-dimensional (3D) hydrodynamic and environmental models. The US Environmental Protection Agency (EPA) later developed it after the Virginia Institute of Marine Sciences initially developed it. This software simulates the flow, transport, and biological processes on the water surface in rivers, lakes, estuaries, reserves, wetlands, and coastal areas. It can simulate the transport of cohesive sediments and non-cohesive discharges nearby and from multiple sources and the transport of pollutants, toxins in water, and sediments. The EDFC Explorer model solves the shallow water equations using a finite difference scheme and a Mellor-Yamada order 2.5 turbulence. It is based on conservation laws of mass, momentum, and thermodynamics.

One of the characteristics of the Garita stream is that its channel is not very wide in some sections, so the mesh generation must have a small cell size. This allows the simulation to represent its behavior; that is, the cells where the Water in the stream must have continuity within them so as not to represent erroneous results at the time of the simulation. When meshing for the area you want to model, it is necessary to consider that it is a repetitive process, which is refined gradually until adequate processing is obtained. This process allows you to identify what type of mesh it is that will best represent the flow behavior at the surface. To do this, it is necessary to establish the limits or borders of the mesh, which is required to verify the coordinate system. In this case, the WGS1984 UTM Zone 14 north system is used in X, Y format.

CVLGrid is an external tool for EFDC that allows building complex 2D curvilinear meshes for hydrodynamic models. This program enables testing different structured meshes to select the one best suits the site’s topography.

One of the most critical steps in the modeling is the calibration; this consists of finding the correct parameters related to the flow behavior, such as roughness, to make the simulated data fit the real ones. The data are measured in the field and taken from an existing hydrometric station inside the channel. This type of calibration in the EFDC is done through *Model Analysis* in the main window, which allows performing a temporal analysis of various parameters, defining and filling the time series comparison, and defining the coordinates and location of the points to analyze. Once this is done, the model uses goodness-of-fit equations, providing a numerical comparison of the simulated and accurate data necessary to adjust the model.

However, for particular situations such as that of the Garita stream, in which there is no record, and in addition, it is a stream immersed in the urban area, modified or rechanneled in some of its sections, in this case, calibration consists of adjusting the topography. Although the topographic survey was carried out using one of the best technologies, the vegetation initially makes noise, so it needs to be adjusted within the channel. After that, the trenches need to be changed to ensure that the water flows inside them and does not remain stagnant.

The topography was modified using the CivilCAD extension of AutoCAD, which allows the import, modification, and export of the mesh. Initially, 700,515 discrete points conformed to the mesh with a separation of 2m between them; however, the calculations are computationally costly due to the number of points. To improve the process and save computational resources, the mesh was set up using 2m in the center of the stream and 4 m in the rest of the mesh.

### Rainfall in the basin

**Figure 12:**
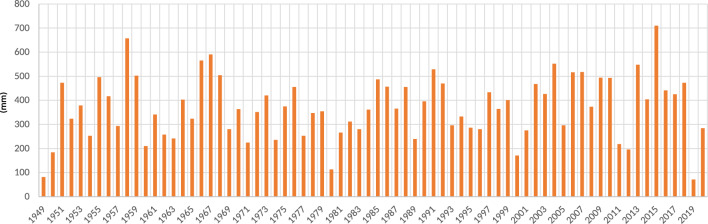
Average annual rainfall in the basin.

The average precipitation of the basin was 391.37 mm from 1949 to 2020. Figure 12 shows the annual rainfall from 1949 to 2019; the historical peaks of rains have increased, with a minimum historical record that during all of 2019, it only rained 50 mm, and four years before, in 2015, a maximum historical peak of 700 mm occurred-evidence of such substantial variations in climate. An exceptional flooding event occurred on August 25, 2016; according to records, it rained 51.6 mm that day. If a probabilistic analysis is carried out, it provides a distribution function to obtain a representation, which presents an estimate of the probability of precipitation in SLP in a given period (Return Period). Historical precipitation data from the climatological station (24069) in San Luis Potosí was recorded to obtain the probability function.

**Figure 13:**
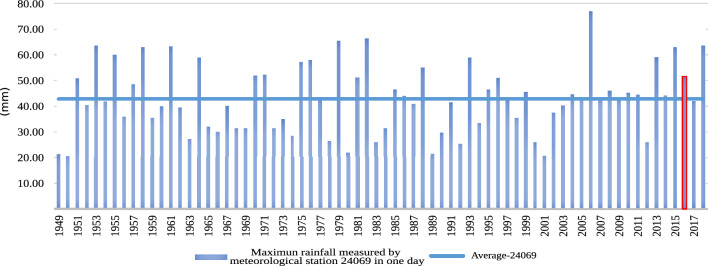
Highest rainfall in a day per year.

**Figure 14:**
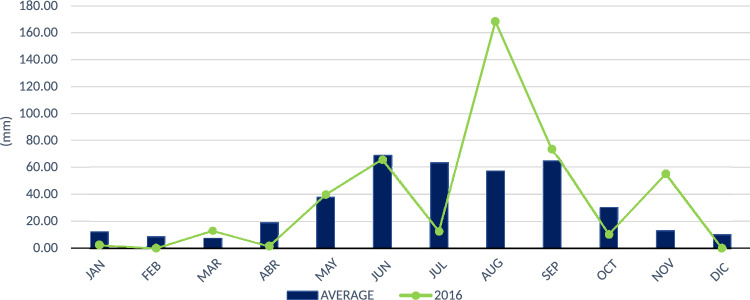
Maximum rainfall measured by weather station 24069 per month in 2016.

According to the graphs depicting the records presented, the rainfall in August (see Table 2) is related to a rainfall period of 5 years. However, according to Fig. 13, the trend of this rain is given approximately every 15 and 20 years, reflecting that cyclical precipitation events do not occur systematically. According to station 24069, August was the month with the most rainfall in 2016 (see Fig. 14), which is more indicative of rains that fell in bursts of an hour. If the aforementioned is accurate, the precipitation recorded in 2016 was almost the precipitation in 1 h and is associated with a return period of 50–100 years, which, as seen with the velocities and depths of the return period of 25 years, is more severe in terms of the response of the runoff and speeds on avenues and streets.

**Table 2:** Rain registers of August 2016, when the flooding occurred.

Date	mm of rainfall	Date	mm of rainfall	Date	mm of rainfall
01/08/2016	0	11/08/2016	4.9	21/08/2016	4
02/08/2016	0	12/08/2016	0	22/08/2016	0
03/08/2016	0	13/08/2016	6.5	23/08/2016	0
04/08/2016	0	14/08/2016	0	24/08/2016	0
05/08/2016	0	15/08/2016	0	25/08/2016	51.6
06/08/2016	0	16/08/2016	5	26/08/2016	0
07/08/2016	0	17/08/2016	2	27/08/2016	0
08/08/2016	28.9	18/08/2016	0	28/08/2016	0
09/08/2016	17.5	19/08/2016	0	29/08/2016	0
10/08/2016	20	20/08/2016	28	30/08/2016	0

**Figure 15:**
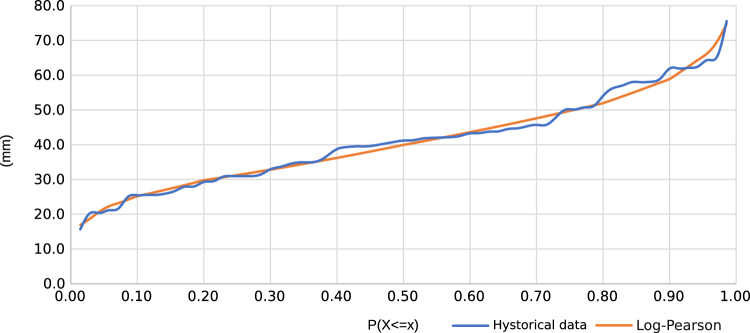
Fit with the Log-Pearson III distribution.

The function that best fits the historical precipitation data is Log Pearson III^29^ (see Figure 15).

**Table 3:** Probability of precipitation.

Return Period	P(X$<=$x)	K	log(X)	P [mm]
2	0.5000	0.0657	1.601	39.9
5	0.8000	0.8550	1.716	52.0
10	0.9000	1.2313	1.770	58.9
25	0.9600	1.6068	1.825	66.8
50	0.9800	1.8352	1.858	72.1
100	0.9900	2.0306	1.886	76.9

According to Table 3, corresponding to the rainfall record, it is related that the heavy rain that occurred on August 25, 2016, corresponds to a rainfall return period of 5 years. As mentioned, this rain pattern shows that cyclical precipitation events do not happen systematically; they happen every twenty-five to fifteen years.

However, the maximum reception recorded in August 2016 by the registered station 24069 can be associated more with a reception that occurred in one hour throughout the month since the existing record is only a daily maximum.

The rainfall of August 2016 is considered an extreme event with a trend of approximately 20 years. This is related to its occurrence with great intensity and infrequency, mainly having environmental and social effects, such as on August 25, 2016.

### Model calibration

One of the most critical steps in any modeling is calibration, which consists of finding the correct parameters related to the flow behavior, such as roughness, to make the simulated data fit the real ones. The data are measured in the field and taken from an existing hydrometric station inside the channel. This type of calibration is done by analyzing the boundary conditions and various model parameters, defining or interpolating different time series at the points of interest. Once this is done, the goodness-of-fit test compares the simulated and accurate data necessary to adjust the model.

**Figure 16:**
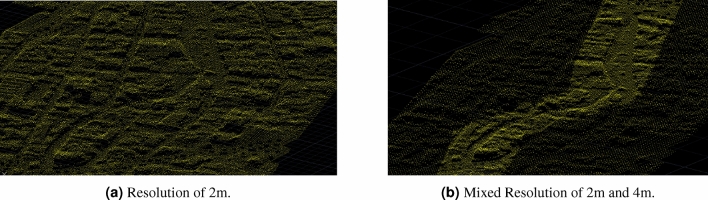
Meshes generated with AutoCAD civil 3D.

As previously mentioned, calibration involves topographical adjustment because no acquired data exists. Although the topographic survey was carried out using one of the best technologies, the vegetation initially makes noise, so it needs to be adjusted within the channel. Then, the trenches must be adjusted to let the water flow inside them so it does not remain stagnant. The topography was modified using the CivilCAD extension of AutoCAD, which allows the import, modification, and export of points. Initially, 700,515 points were exported to the EFDC with a 2 m separation between them. However, due to the number of these, the calculation is slow, and the definition in the center of the stream is poor. Later, to improve the process, 310,143 points were made in 2 m in the center and 4m in the right and left banks, in addition to *cleaning* the section of the stream. The comparison of the mesh resolution is shown in Fig. 16.

**Figure 17:**
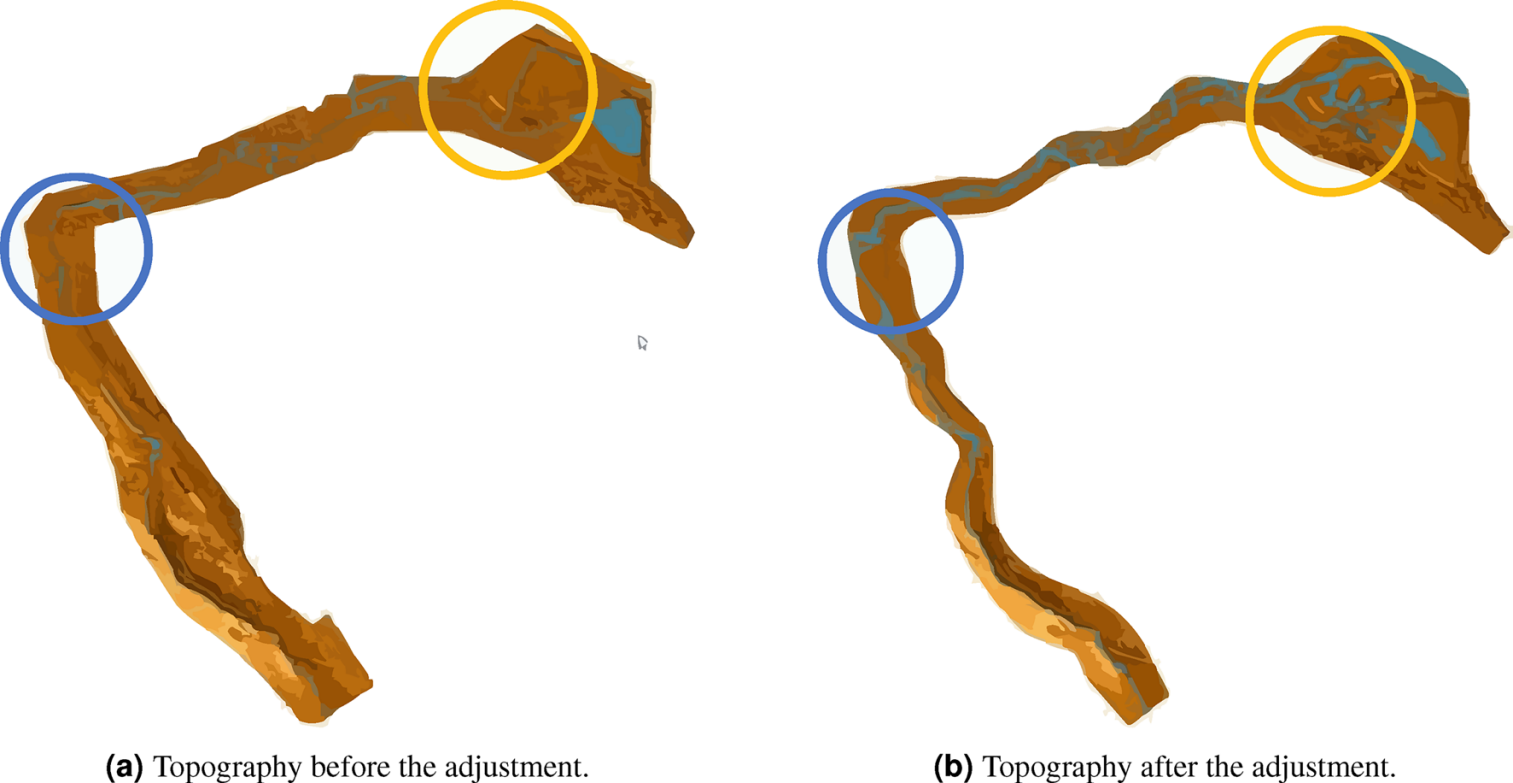
Comparison of topographic resolution. The circles show zones that require manual adjustment for flow continuity. Images generated with EFDC Vizualizer (https://www.eemodelingsystem.com/).

During the topography adjustment process, the bridges are considered to represent them in the simulation. As shown in Fig. 17, this adjustment involves changing the input and outlet cells to keep the flow continuous. It is essential to check the elevation of the cells designated as culverts and those before and after them so that water can flow freely through the culverts. The program can also predict how the bridges will behave.

## Results

The SWMM, HEC-RAS 2D-3D, and EDFC models were used for the return periods established since the similarity of the results obtained validates the results presented. Nevertheless, the results obtained with the EDFC are presented mainly because they have better resolution. The return periods of 2, 5, 10, 25, and 50 years were used to establish the simulation scenarios, which are recommended in the literature for hydrological studies; these allow them to be used in the future for the subsequent design of works that will enable reducing the damage that is generated with the overflowing of the stream. The result of the stream flow below the bridges is carried out in different scenarios, simulating the hydrograph of the peak flow in the same periods. An additional scenario with a stream of 2 $\hbox {m}^3$/s is simulated; this scenario is to ensure a free flow.

**Figure 18:**
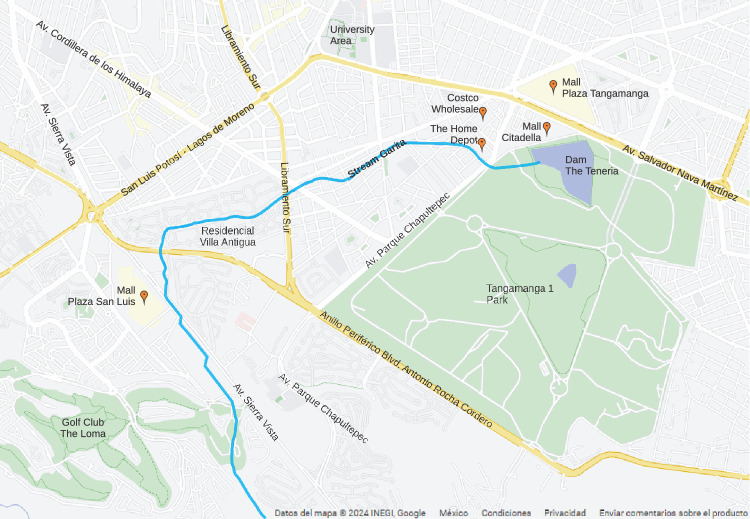
Places of special interest.

The simulations of the different scenarios with EFDC consider the conditions of the entire stream, allowing a better spatiotemporal analysis. After running the simulations for 12 h, the hydrographs were added at various return periods with a base time of 8:24 h. This way, the overflows could be seen acting at the different locations described in the analysis of each simulation (see Fig. 18).

Table 4 shows the different flow rates used for each return period.

**Table 4:** Flow rates used for the different scenarios.

Return period (years)	Flow rate $\hbox {m}^3$/s
2	8.28
5	12.56
10	16.14
25	21.24
50	25.33

### Return period of two years

**Figure 19:**
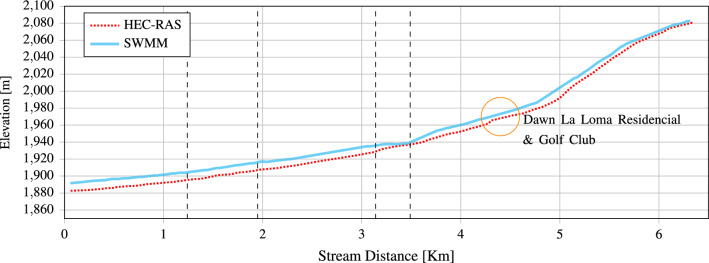
Comparison of SWMM and HEC-RAS profiles for a return period of 2 years. Compared with the EDFC HEC-RAS gives a better estimation.

The first simulated scenario is for the two-year return period, introducing the hydrograph with a peak flow of 8.28 $\hbox {m}^3$/s as input to the model. Since the stream is a transient current, it occurs only in the rainy season, simulating it almost entirely. In the initial scenario, stream overflows are seen. Simulations conducted in SWMM indicate that most stream sections are inadequate for flow passage. These findings are consistent with the results obtained by HEC-RAS and EFDC Explorer. In this scenario, according to the profiles compared in Fig. 19, the flow covers the section of the stream in most areas, mainly from the passage to the closed street in front of Plaza San Luis (km 3.49) until its exit towards the peripheral ring (km 3.14) at the first bridge of Av. Cordillera de los Himalayas (km 1.95–1.93) and at Av. Niño artillero (km 1.24–1.23). It is to be considered that unidimensional models simplify the system by assuming flow only along one axis or direction. This can lead to inaccuracies, especially in systems where flow behavior is inherently bi-dimensional. The HEC-RAS and SWMM are set up with the same hydraulic flow; according to the EDFC, the HEC-RAS is more precise. Because they solve the one-dimensional shallow water equation, the only way they can conserve mass is by increasing the elevation.

**Figure 20:**
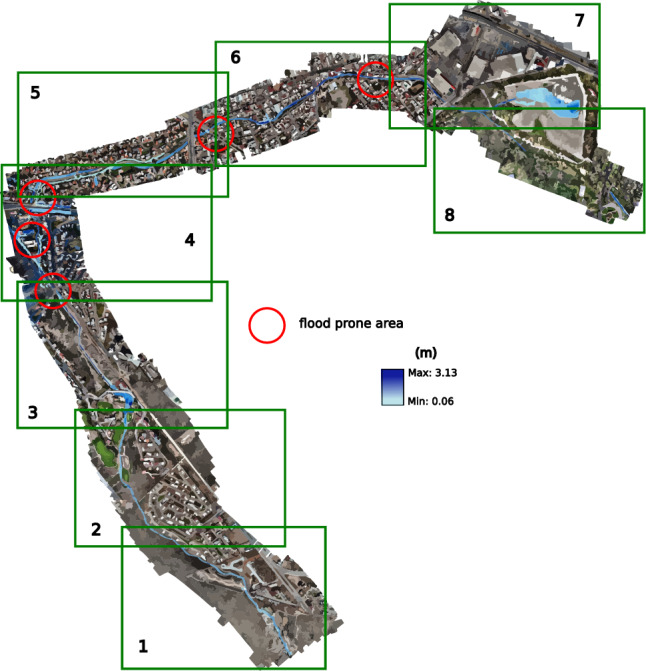
Snapshot at the end of the two-year return simulation period showing the flood-prone zones. Images generated with EFDC (https://www.eemodelingsystem.com/ee-modeling-system/efdc_explorer/overview).

**Figure 21:**
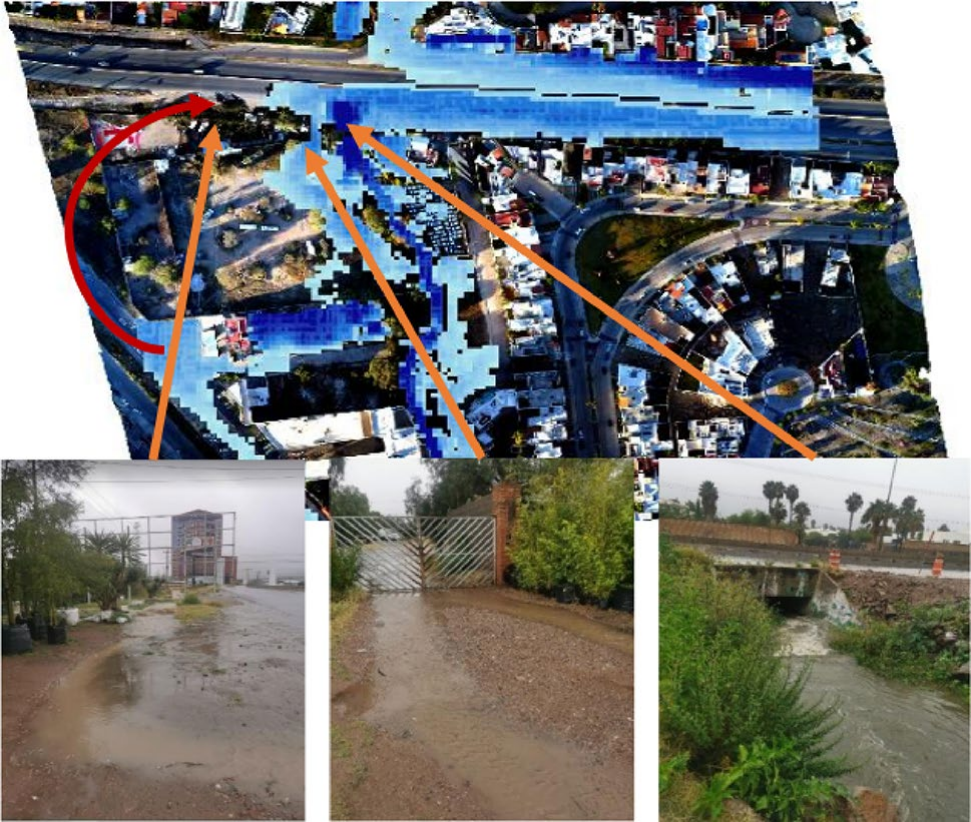
Runoff from Sierra Vista Avenue towards the ring road (Anillo Periférico) in August 2020, match with the prediction in the simulation of the two years return period.

However, EFDC improves the solution by considering the entire channel and urban area. Figure 20 displays the simulation produced using EFDC, and Fig. 21 displays the runoff that the simulation predicted.

### Return period of five years

In this second scenario, with an inlet hydrograph showing a peak flow of 12.56 $\hbox {m}^3$/s, there is a more significant overflow in the areas already observed in the first scenario. Still, a considerable amount of water flows to the streets. Although this scenario is associated with the events of 2016, the stream does not overflow on the Chapultepec Avenue bridge, crossing Tangamanga I. However, the surge towards secondary streets is more significant than in the first scenario, and flood points increase. Very similar results in the 1D modeling are no longer projected for this scenario; however, more remarkable similarities in the modeling were found in the 2D comparison.

**Figure 22:**
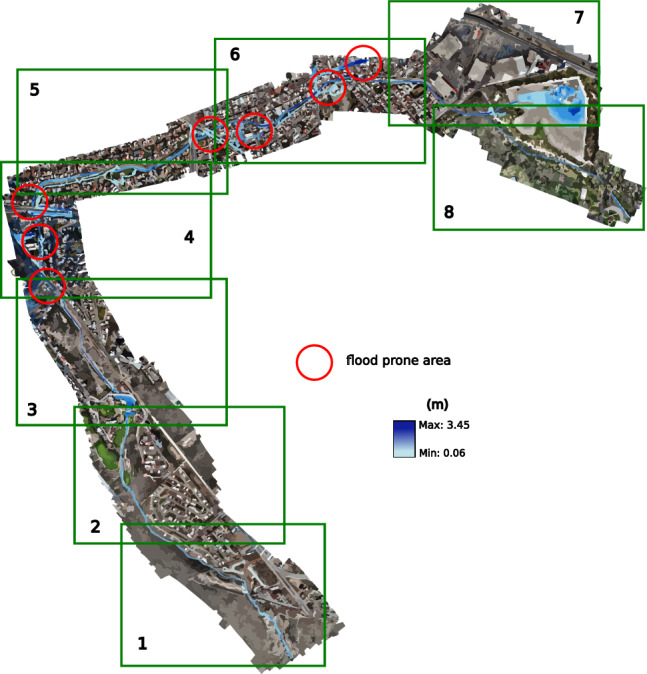
Snapshot at the end of the five-year return period simulation showing the flood-prone zones. Images generated with EFDC (https://www.eemodelingsystem.com/ee-modeling-system/efdc_explorer/overview).

In the case of modeling in HEC-RAS, the results were obtained using a permanent flow, without using the spatio-temporal behavior of the flow. The projection of EFDC results was performed at 3:40 h after the start of the hydrograph. Based on these results, exits are similar to the modeling in HEC-RAS and EFDC; for this reason, only results obtained with EFDC are presented (see Fig. 22).

### Return period for ten years

For the third ten-year scenario, with a peak flow in its hydrograph of 16.14 $\hbox {m}^3$/s, the overflow is not only more evident in the points already observed in the previous scenarios, but in this one, the overflow in the avenue Chapultepec begins to become evident almost at 6:20, from the beginning of the inlet hydrograph.

**Figure 23:**
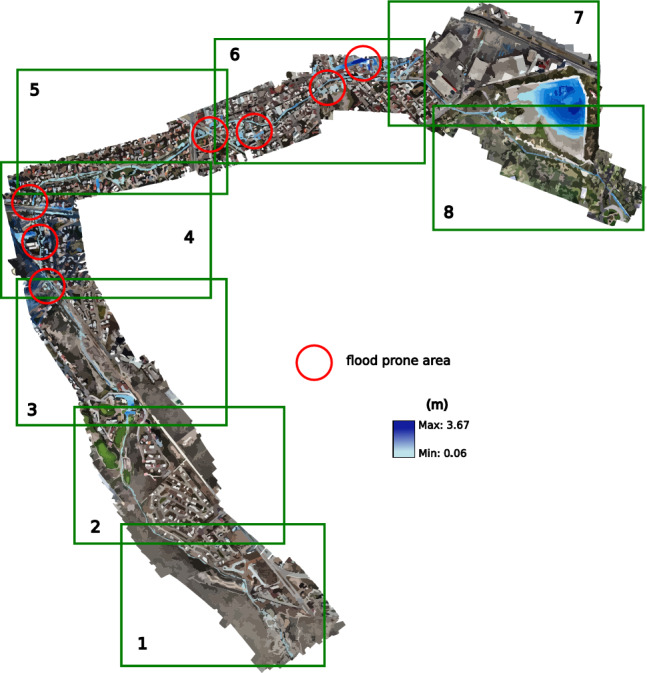
Snapshot at the end of the ten-year return simulation period showing the flood-prone zones. Images generated with EFDC (https://www.eemodelingsystem.com/ee-modeling-system/efdc_explorer/overview).

From this case, the overflow of the Arroyo Garita becomes more evident in the point of conflict raised in this work. However, there is no current over the avenues like in 2016. In this scenario, the conflict points are similar to the previous period, causing less than 5 cm of flooding on Av. Chapultepec. The flood-prone areas can be seen in Fig. 23.

### Return period for twenty-five years

**Figure 24:**
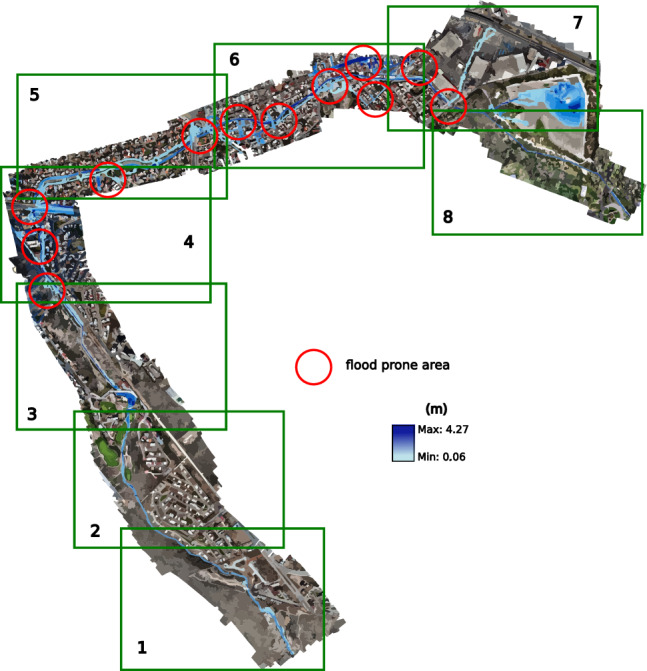
Snapshot at the end of the twenty-five-year return simulation period showing the flood-prone zones. Images generated with EFDC (https://www.eemodelingsystem.com/ee-modeling-system/efdc_explorer/overview).

The fourth 25-year scenario has a peak flow of 21.24 $\hbox {m}^3$/s in its inlet hydrograph. In addition to the previous overflows, which in this case become more extensive, an overflow begins to occur at the first bridge over the stream, and greater flow over Chapultepec Avenue towards Dr. Salvador Nava Avenue. The results for this return period are projected in Fig. 24, where they have presented the conflictive points and a better visualization at different times of the simulation; the first section is presented at 1:50 h, shortly after the entry of the peak flow, the second section at 2:10, sections 3 and 4 in 2:50, the 5th and 6th in 3:20, and the last 2 sections at 7:50 h, where an invasion already occurs on Dr. Salvador Nava Avenue. The results of this scenario show more of what happened in 2016, although there could be greater flow on the avenue due to previous overflows in the earlier scenarios.

### Return period for fifty years

**Figure 25:**
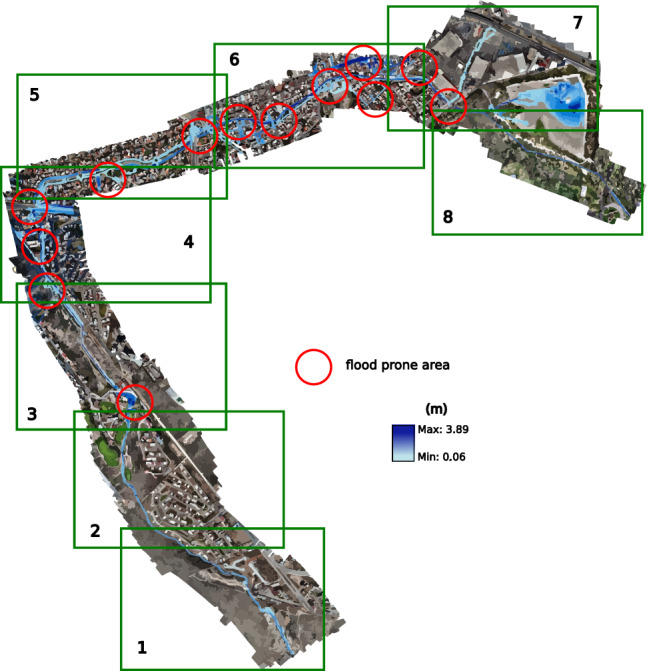
Snapshot at the end of the fifty return simulation period showing the flood-prone zones. Images generated with EFDC (https://www.eemodelingsystem.com/ee-modeling-system/efdc_explorer/overview).

In the 50-year scenario, the maximum flow rate seen in the inflow hydrograph is recorded as 25.33 $\hbox {m}^3$/s. In this particular scenario, when certain areas within the city experience flooding, similar to the prior situation, the primary focus is on the flow passage at the Loma dam and the initial bridge, as they exhibit the highest levels of impact. According to section 2, simulated in Fig. 25, the dam exhibits sufficient capacity to accommodate the water flow without experiencing overflow. Similarly, the bridge located within the residential area, which serves as a partition for the dam, also possesses the necessary capacity. Furthermore, the flow is directed across the initial bridge of the stream, where a minor overflow may be noticed on a single side of the stream, rejoining the stream without traversing the planned roadways of the newly constructed residential development.

## Simulations validation

There are three different ways to validate a model:

- Comparing the results obtained from the model with the results of theoretical cases that propose an analytical solution allows for determining the precision of the results.
- Solve the same problem using other models with similar characteristics to compare results.
- Through comparison with measurement campaigns at strategic points of the study zone.

In this case, there are different overflow events, such as the year 2016, related to a 5-year return, and knowing, according to witnesses, of the specific areas where the stream overflowed, validation was performed using the information in the field. Additionally, we compare results using other numerical models, such as SWMM in 1D and HEC-RAS in 1D and 2D.

**Figure 26:**
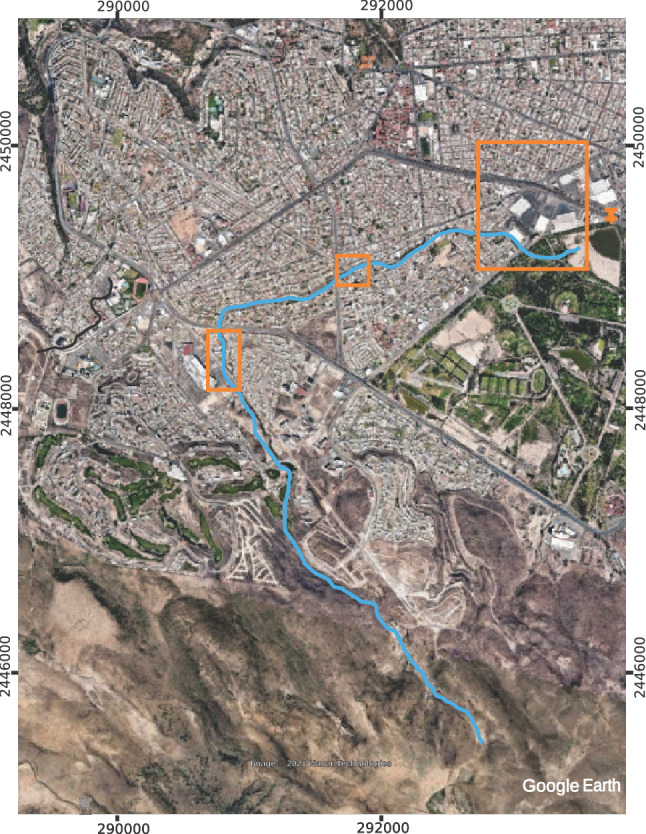
Overflow zones used for validation.

**Figure 27:**
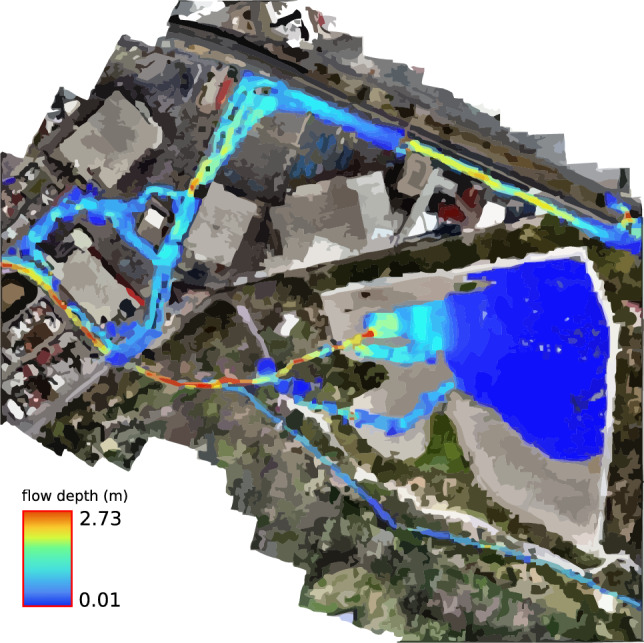
Possible overflow towards Av. Chapultepec of Anillo Periférico, 25-years return period. Images generated with EFDC (https://www.eemodelingsystem.com/ee-modeling-system/efdc_explorer/overview).

Given the absence of historical data about the stream’s flow, the model is validated by comparing simulations across various scenarios and models. This comparison mainly focuses on sections where overflow occurs, as evident in the initial two models. Notably, the 2-year return scenario serves as an indicator for such overflow. The validation process also analyzes Av’s flow behavior and overflow. Chapultepec, precisely where it intersects with Tangamanga I park. The effects of this validation become noticeable after a decade has passed. The overflows occurring at various locations upstream of the stream in the initial scenario are verified using testimonies provided by the individuals residing in the area experiencing the overflow. The private streets are located in front of Plaza San Luis and Av. The Himalayan mountain range is subject to an annual occurrence where the stream surpasses its capacity, leading to discontent among the residents residing in the affected regions. The depicted areas can be observed in Fig. 26, together with the precise positioning of Av. Salvador Nava. The intensity of the overflow in Salvador Nava increases significantly in scenario 4, which has a return duration of 25 years. This scenario exhibits speeds above 1 m/s on the Avenue, resulting in an overwhelming situation for pedestrians due to the flow’s high volume and velocity. These findings are depicted in Fig. 27.

This scenario validates the simulations with variable depths of up to 1 m along the avenue, showing more speed and depth in front of Citadella Square, similar to the evidence from 2016. In addition, it observes that there is no flow into the stream unless there is precipitation.

**Figure 28:**
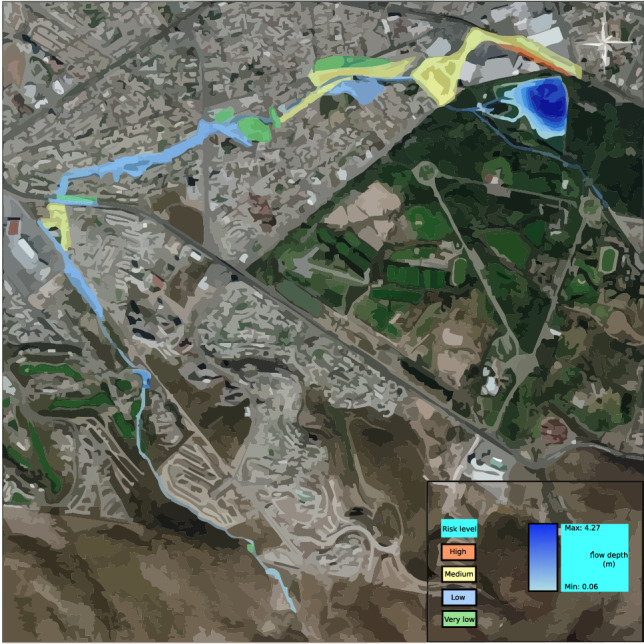
Risk map over the Garita stream, according to the CONAGUA diagram. Images generated with EFDC (https://www.eemodelingsystem.com/ee-modeling-system/efdc_explorer/overview) and edited with Inkscape (inkscape.org).

**Figure 29:**
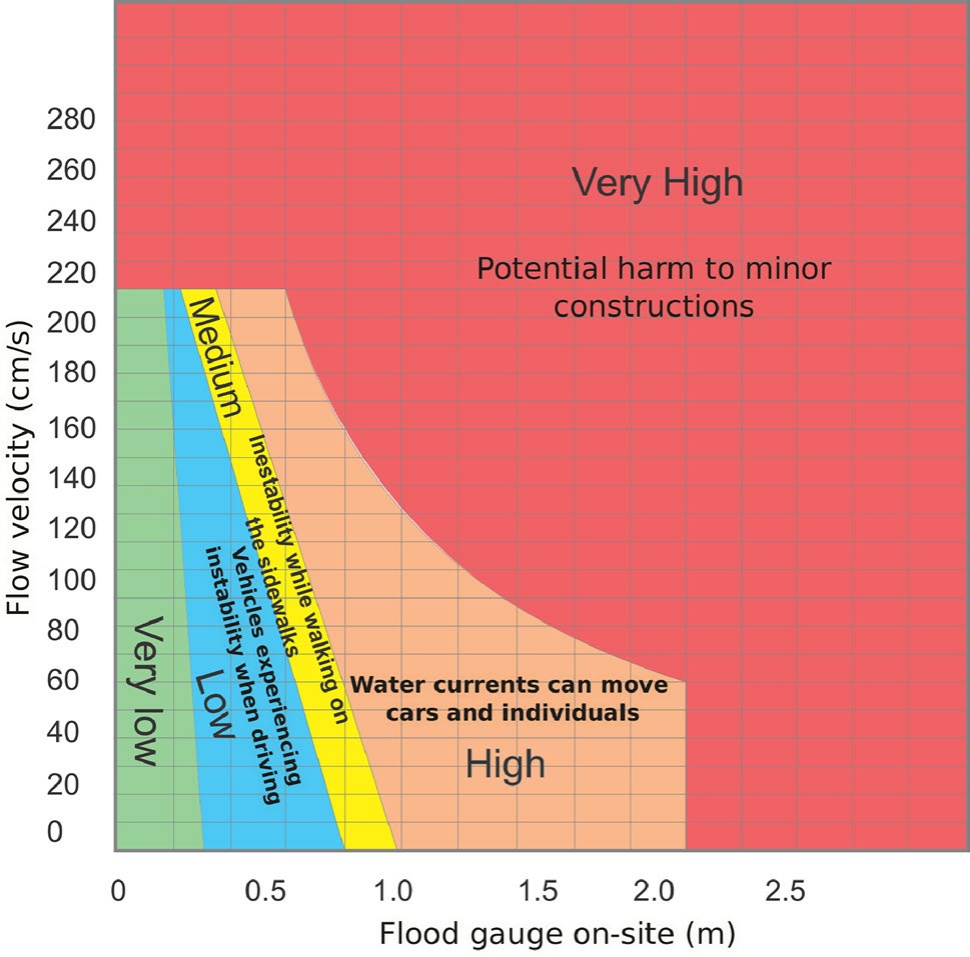
The severity diagram obtained from the National Water Commission (CONAGUA) (https://www.gob.mx/conagua).

The risk map was constructed by evaluating the depths of the 25-year return period, as depicted in Fig. 28, using the recommendations and risk map graph provided by CONAGUA (2014) (Fig. 29). This was done while considering the speeds and flood braces at the location below 3 m/s.

### Proposals for potential prevention and resolution strategies

The overflow of the Garita stream causes floods in the Garita basin area, which includes points along the creek within the urban area. Poor maintenance of the stream in unlined areas, ineffective storm drainage, and an inefficient section of the stream at various return periods cause flooding in an already-developed area. Due to the impervious surface that urbanization has created, water accumulates in streets and lower areas. Different options can be implemented in the city to prevent and solve overflows and flooding in the urban area, from the simplest, such as increasing the edges through walls and the construction of sewage systems, to sustainable urban drainage systems, improving bridge sections, and water infiltration, among others. Since there is no space within the urban area to expand the stream, it is necessary to think about implementing options other than the usual ones, such as water infiltration at critical points or sustainable urban drainage systems in all conflict areas, which, in addition to minimizing runoff on the streets, is helpful to the same population by collecting overflowing water.

The above can be implemented at other points within the urban area, as well as storm drains or expansion of the sewage system, since there is no space for increasing the section of the stream nor infrastructure that serves as a retention vessel, such as This is the case of the Loma Residencial Dam. In the case of the private area in front of Plaza San Luis, as there is a green area on the right bank of the stream, a wall or edge greater than 1 m can be implemented for depths of up to 0.89 m, by the results, which prevents access to the impervious surface of the private street.

**Figure 30:**
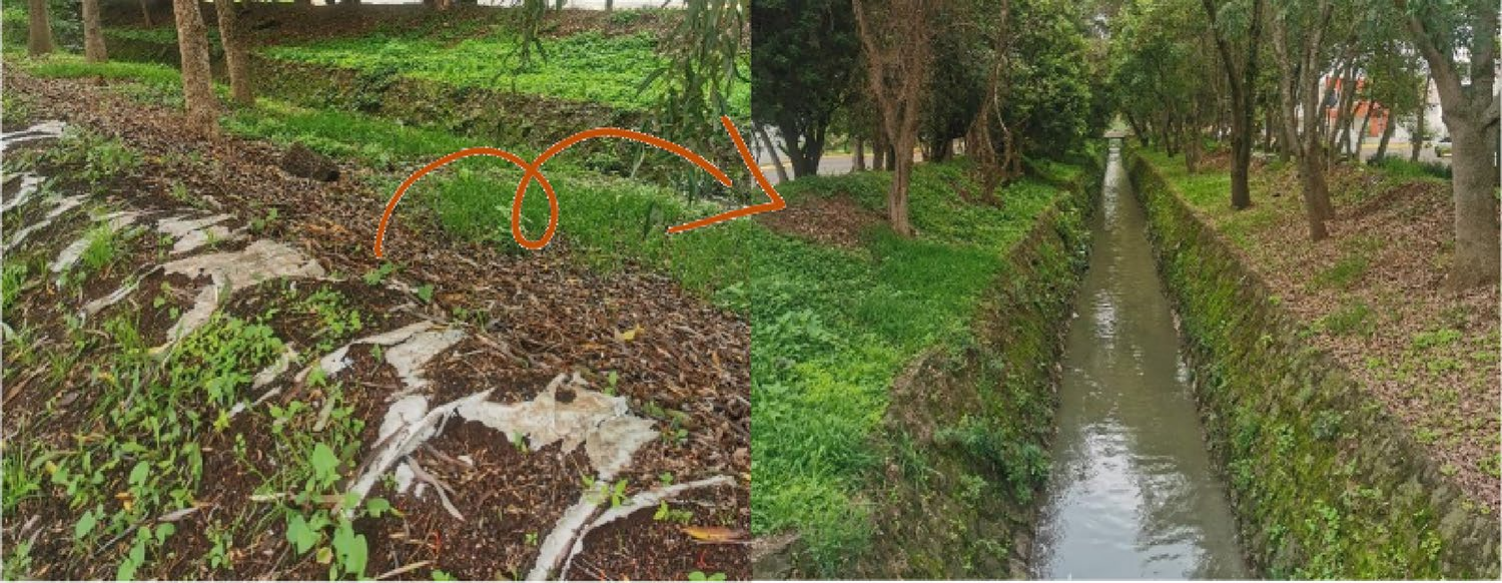
The concept of edge of land has been applied in the case of the Campestre la Huerta subdivision in Morelia, Michoacán.

These edges can be implemented from earth or earth coasts, which also do not destroy the green area and mix with the vegetation, thus avoiding erosion, as shown in Fig. 30, which is a case implemented a few years ago in a set residential area that was constantly flooding, thus preventing the river from overflowing.

Most of the overflows in the urban area are caused by bridges over the stream, which have sections that are insufficient for water passage. From the first stage, flowing through the streets, as in the Peripheral Ring case, the flow is directed towards the Villantigua residential complex. In the case of bridges, if possible, their area must be increased, allowing the flow to pass through; however, when the section is modified, modeling is required to verify the passage of the flow through the unit. Otherwise, it would continue to exist. In addition, the stream requires maintenance throughout its section since vegetation growth reduces its area; this also influences photogrammetric surveys since this is captured and must be fixed as part of the calibration.

## Conclusions

Identifying flood-prone areas is paramount as the city’s urban expansion continues. The Garita Stream basin is an area invaded by urban expansion. Although it is not a very large area, a detailed analysis is required to understand the effects of rainfall within the city. The lack of up-to-date information on rainfall, use, type of soil, and topography of the area led to the acquisition of information in the field. Like other branches, the topography is different and multidisciplinary. With the advancement of technology, it is of great importance to update methodologies and disciplines, such as geodesy and photogrammetry, that may be involved in carrying out a study more precisely, faster, and cheaper. As this is a temporary stream, it does not have hydrometric information recorded for modeling, so for this topography, details are necessary to know the conditions of the stream and the urban area. Using a UAV for a topographic survey and GNSS receivers present greater precision than traditional surveys for modeling projects, in addition to a detailed overview of what is found in the area to be studied in a shorter time. Once the field information from the surveys for the stream and right and left banks has been managed, post-processing of the information is required to make the topography more precise and, in addition, to be able to project it in space to be visualized in large scale through an MDE, thereby giving a more excellent overview of the importance and detail of the situation of the stream. The program used for the projection of the topography using MDE allows the classification of objects within the scenario captured by photographs, making different MDE with which the passage of flow and overflow within the urban area can subsequently be represented. The use of updated equipment for the topographic survey is efficient; however, as the urban area invades the stream, a detailed study of the existing hydraulic structures is required for its later introduction. In the programs and the correct representation of the flow passage in the channel to carry out the hydrological study, the lack of information in the area and even the empty months and years in the existing meteorological stations makes the estimates less precise since, although a station is close to the basin, the data may have Significant variations are seen in the records from stations 24069 and 24111 within the city. In the same way, as there are missing data in the stations and when carrying out different statistical processes for a hydrological study, the solutions are less precise than accurate and historical data, affecting the quality of the study, which depends directly on the existing information. Therefore, since there are different methodologies, from testing existing data to estimating inlet flows for the numerical model, different methodologies that have been tested in the same area or places with climatological characteristics and topographical images similar to the study area are used, thereby minimizing errors and making estimates as close to reality as possible. Hydrodynamic modeling using computational equipment is a tool for solving complex equations that describe the behavior of free surface flow in 1D, 2D, and 3D to achieve reliable simulations according to the results obtained in the different dimensions when not existing recorded data for calibration with accurate data. The three modeling programs used require data that allow us to begin the solution of the governing equations through inlet flow and boundary conditions coming from the hydrological study, surface roughness, and the post-process of the topographic survey. The 1D stream profile model, using the SWMM and HEC-RAS programs, requires topography by sectioning the stream, providing with its results a pre-panorama of the sections of the stream that are conflictive with the flow passage in the different stages. By matching the results of the two programs, the reliability that the input data was entered correctly and using a more complex program. HEC-RAS, in addition to showing results in 1D, provides 2D results that are later used for comparison with the 3D model. Its interface allows the introduction of the MDE, which makes it possible to generate more sections and, with these, make the simulation more precise. In addition, within this program, it is easy to introduce the existing infrastructure, detailing each bridge over the stream. Finally, with the EFDC program, although it involves more configuration of the input data, the results are more reliable and visible since it shows the spatio-temporal behavior of the flow. The projected results of solving equations in a discrete scheme are more reliable when considering the section of the stream to along this, without cutting it, as are the results of the previous programs. In this case, calibrations must be carried out with trial and error simulations, detecting obstructions other than the existing infrastructure, modifying these to pass the flow, and changing cells through which there are bridges. Like calibration, validation is done by observing and comparing the different models and results with the inhabitants’ experience in the conflict points. The different scenarios in the three models show similar and possible results according to the existing conditions. The few discrepancies between these may be due to the way the governing equations are solved and the introduction of boundary conditions such as topography, as in the introduction of sections, where the existing characteristics between them are lost. The simulated results show that the stream does not have the capacity for the flow to pass at several points, starting with Av. Sierra Vista, where the section has been expanded to Av. Chapultepec is the same case as the first; however, possibly due to the section and conditions of the stream, these become insufficient. From the first scenario to the last one modeled within this work, the stream presents overflows, with peak flows that increase from 8 to 25 $\hbox {m}^3$/s in the last one scenery. The first scenario shows minor overflows, such as flooding not exceeding 30 cm. However, when the following scenarios are projected, they become more severe, increasing the stream’s depth and overflowing points. Although the scenarios of 5 and 10 years present an overflow on Av. Chapultepec towards Dr. Salvador Nava, this flow is not as important as it was in 2016, the closest scenario and similar to the flood being the following 25-year scenario, also showing depths greater than 1 m in some points, speeds that can drag the residents, as happened. Through the projections, the flow accumulation is not only due to the emerging flow of av. Chapultepec, but the overflowing water is shown at more than one point, being able to join these, the overflowing water of the Peripheral Ring, which connects to Av. Chapultepec. However, since the recent topography of this is not available, the different runoffs that could have flowed into the avenue cannot be verified within this work. Since the overflow on Av. Chapultepec did not appear in the results after five years. As had been assumed within the hydrological study, initially due to the distance at which the recorded data is located from the basin and the lack of meteorological and hydrometric information on the stream; however, since S. L. P. is a semi-arid city, this record can be associated with precipitation that occurred in a shorter time, similar to a period between 50 and 100 years, resulting in a more dangerous scenario.

### Supplementary Information

Supplementary Video 1.

Supplementary Video 2.

Supplementary Legends.

## Data Availability

C.C-C. can provide all the datasets used in this research under a reasonable request.
