# Supplementary material for: Flood risk assessment of the Garita River in the urban zone of San Luis Potosí City, by hydrodynamic modeling
Source: Sci Rep. 2024 Jul 10;14:15891. doi: 10.1038/s41598-024-66743-1 (PMC11237141; doi:10.1038/s41598-024-66743-1)
Supplement: Supplementary file 3 — Supplementary Legends. [file 41598_2024_66743_MOESM3_ESM.pdf]

San Luis Potosí, Mexico, August 26, 2016

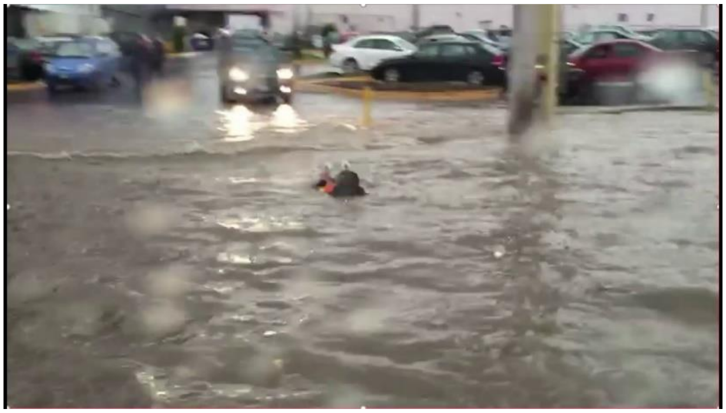

Video 1. A man carried away by a strong current on the avenue

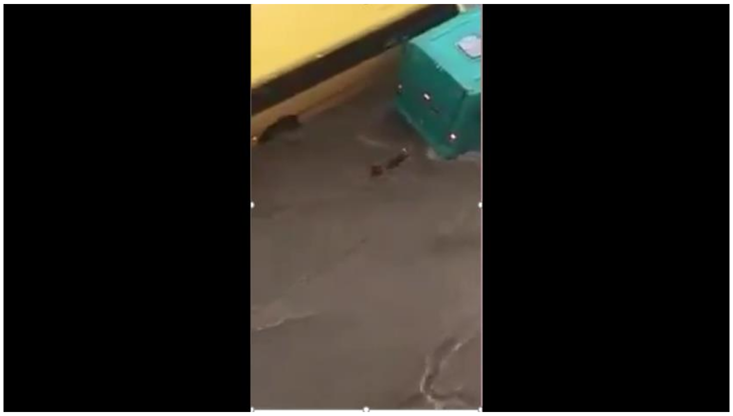

Video 2. Continuation of the video when he can stop at the back part of a bus.

Due to the heavy rains in the region, a man who tried to cross Salvador Nava Avenue in front of Citadella was dragged by the current for several meters. He passed under a city truck, and after several meters, he managed to save his life thanks to the fact that he ran into a car, which prevented him from continuing to advance.

It is presumed that a minor was swept away by the current in the Lomas subdivision when he was walking down the street with his father, a bricklayer, to go home. The Public Security Department of the State and municipal police implemented an operation to locate him.

https://www.elfinanciero.com.mx/bajio/fuertes-lluvias-causan-inundaciones-en-san-luis-potosi/

https://vanguardia.com.mx/noticias/nacional/corriente-arrastra-hombre-en-calles-de-san-luis-potosi-tras-fuertes-lluvias-LOVG3245718
